# Terahertz Metasurface with Four-Degree-of-Freedom Geometric Encoding for Broadband Multichannel Fingerprint Sensing

**DOI:** 10.3390/nano16171059

**Published:** 2026-08-26

**Authors:** Jianming Meng, Wei Hao, Tianlu Wang, Yanpeng Shi, Weiqi Xu, Mengya Pan

**Affiliations:** 1 Department of Electronic and Communication Engineering, Shandong College of Electronic Technology, Jinan 250200, China; sdcet_haowei@163.com (W.H.); wtl9808@163.com (T.W.); 2 School of Integrated Circuits, Shandong University, Jinan 250101, China; ypshi@sdu.edu.cn (Y.S.); 202200202005@mail.sdu.edu.cn (W.X.); 202432401@mail.sdu.edu.cn (M.P.)

**Keywords:** metasurface, THz fingerprint sensing, four-degree-of-freedom geometric encoding, multichannel detection, L-hydroxyproline

## Abstract

Terahertz (THz) fingerprint spectroscopy enables label-free identification of molecular vibrational signatures, but trace biomolecular absorption is too weak to be reliably resolved in free-space measurements. To address this limitation, we propose a four-degree-of-freedom geometrically encoded THz metasurface for broadband multichannel fingerprint sensing. The substrate-free self-supporting aluminum structure incorporates four independently tunable geometric parameters: gap angle *θ*, outer ring radius *R*, scaling factor *S*, and ring width *W*. By regulating these parameters, multiple resonance-tuning pathways are established, enabling designable multiband spectral coverage over 0.6–1.4 THz and flexible matching with the fingerprint bands of L-hydroxyproline (L-HYP). Numerical simulations show that the metasurface achieves a refractive-index sensitivity of 512.66 GHz/RIU with a linear fitting coefficient of *R*^2^ = 0.99708 and a mean *Q* factor of 4.72. For biomolecular fingerprint sensing, the encoded resonances overlap with the L-HYP absorption bands near 0.73 and 1.17 THz, producing AIT-like spectral modulation and envelope-derived attenuation enhancement. Compared with an unstructured analyte reference, the valid 0.73 THz readout gives enhancement factors of 5.76 and 7.09 for the *R* and *S* channels, respectively, while the four encoded channels provide enhancement factors of 3.53–4.45 at 1.17 THz. This design provides a compact strategy for broadband multichannel THz fingerprint enhancement, offering a promising route for monitoring collagen-metabolism-related biomarkers and advancing label-free biochemical sensing, fibrosis-related molecular screening, and integrated broadband THz detection.

## 1. Introduction

Terahertz (THz) fingerprint spectroscopy probes low-frequency molecular vibrations, rotations, and intermolecular weak interactions, and therefore offers a label-free route for biochemical identification, trace detection, and disease-related molecular screening [1,2,3,4]. In contrast to label-based assays or purely refractometric readouts, THz spectra can directly reflect intrinsic molecular absorption features. However, under trace-sample conditions, the interaction between free-space THz waves and minute amounts of biomolecules is intrinsically weak, so fingerprint signals are easily obscured by background noise, system loss, and sample-preparation variations [5]. Enhancing THz–molecule interactions while preserving molecular-specific spectral information is therefore a central challenge for practical THz biosensing.

Metasurface-enhanced THz sensing provides an effective strategy to address this weak-interaction problem. Subwavelength resonant elements can localize electromagnetic fields near the analyte and convert subtle dielectric or absorptive perturbations into measurable frequency shifts, linewidth variations, amplitude modulations, or transparency-window changes [6,7,8,9,10,11]. Recent studies have demonstrated THz metasurfaces for amino acids, tumor biomarkers, proteins, pathogens, and other biological targets, confirming their value for improving sensing sensitivity [12,13,14,15,16,17,18]. Nevertheless, many reported sensors still rely mainly on refractive-index shifts around a single resonance. Such single-channel responses can be affected by nonspecific adsorption, environmental drift, and background fluctuations, which increases the risk of false-positive identification. Moreover, high-Q resonances improve spectral resolution but are usually narrowband, whereas biomolecules may exhibit more than one characteristic fingerprint feature. Although a single clear fingerprint peak can provide molecular information, cross-validation using multiple characteristic responses can improve recognition reliability. Therefore, improving only the single-point sensitivity or Q factor is insufficient for broadband molecular fingerprint recognition and reliable multichannel discrimination [19,20].

To bridge the gap between narrowband field enhancement and broadband fingerprint recognition, THz metasurface sensing is evolving toward multichannel spectral sampling and discrete space–spectrum encoding. Externally tunable metasurfaces and pixelated array strategies have expanded the accessible resonance range and promoted the development of broadband THz spectroscopy [21,22,23,24,25,26,27,28,29]. However, several limitations remain. Large-angle scanning or external tuning inevitably increases system complexity, while simple pixel stitching is constrained by chip area and spectral sampling density. Therefore, a compact THz platform that integrates broadband coverage, facile fabrication, and multi-degree-of-freedom resonance encoding is still needed to support multichannel fingerprint enhancement and cross-validation of molecular absorption features.

L-hydroxyproline (L-HYP), a derivative of L-proline, is an important collagen-related amino acid. Because hydroxyproline is a relatively specific constituent of collagen, its quantification is widely used to estimate tissue collagen content and concentration [30]. Excessive collagen accumulation is a hallmark of fibrotic remodeling, particularly in progressive renal fibrosis [30]. In addition, solid L-HYP exhibits distinct THz absorption features, with characteristic peaks reported near 0.73 THz, approximately 1.05–1.17 THz, and 1.82 THz, these literature-reported THz-TDS features and their vibrational assignments provide the spectral basis for selecting L-HYP as a representative model analyte [31,32,33,34]. Thus, L-HYP is biologically meaningful and spectroscopically suitable for evaluating broadband multichannel THz fingerprint-enhanced sensing.

In this work, we propose a THz metasurface with four-degree-of-freedom geometric encoding for broadband multichannel biomolecular fingerprint sensing. The structure incorporates four independently adjustable geometric parameters, namely the gap angle *θ*, outer ring radius *R*, scaling factor *S*, and ring width *W*, enabling fine encoding of resonance frequency, spectral response, and multiband spectral coverage. Unlike single-parameter gradient structures, the proposed strategy establishes multiple independent resonance-tuning pathways within the same resonant architecture by separately varying *θ*, *R*, *S*, and *W*. Encoded resonators selected from these tuning pathways can then be spatially arranged to form a discrete multichannel metasurface, allowing different resonance channels to cover the target molecular fingerprint region. By combining four-degree-of-freedom geometric tuning with discrete encoded-resonator arrangement, the metasurface produces stable simulated responses over 0.6–1.4 THz and covers the key fingerprint absorption bands of L-HYP. The self-supporting, substrate-free aluminum configuration promotes direct contact between analyte molecules and localized electromagnetic fields, and simplifies fabrication. This design integrates broadband coverage, multichannel fingerprint response, AIT-like molecular coupling, and four-degree-of-freedom geometric tunability into a single sensing platform, providing a compact and manufacturable route for label-free THz fingerprint-enhanced detection.

## 2. Materials and Methods

**Figure 1:**
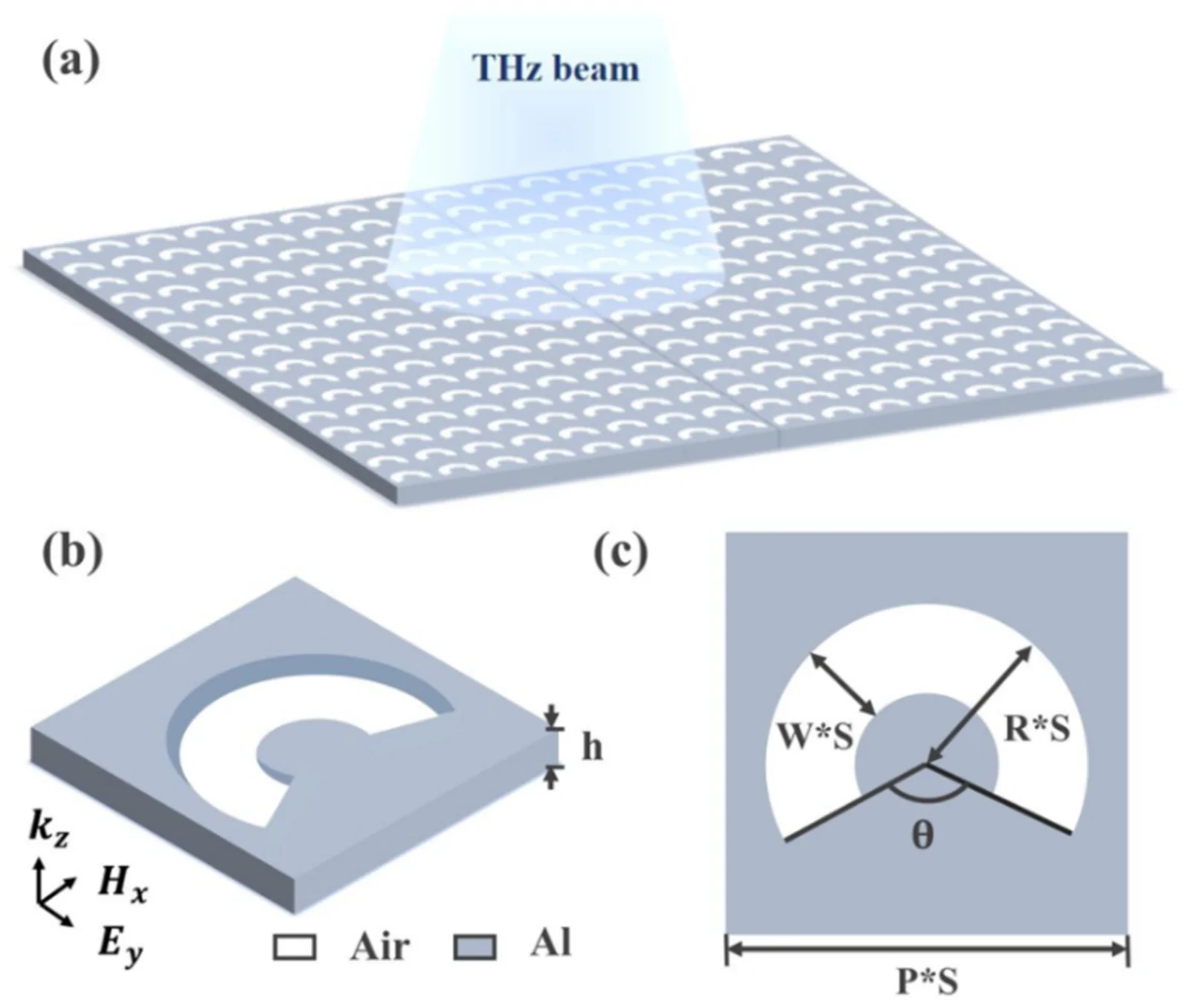
Structural design of the proposed terahertz metasurface. (**a**) Overall schematic of the metasurface device. (**b**) Schematic of a single unit cell with four-degree-of-freedom geometric encoding. (**c**) Detailed structural parameters and dimension definition of the unit cell. The asterisk (*) denotes multiplication by the scaling factor *S*.

As illustrated in Figure 1a, the proposed terahertz metasurface sensor adopts a self-supporting, substrate-free aluminum configuration and operates under normal incidence of THz waves. The absence of a dielectric substrate avoids additional substrate-induced loss and allows the analyte overlayer to directly interact with the metallic resonant structure, which can effectively enhance light–matter interactions and is suitable for label-free detection of trace biomolecules and multi-band broadband sensing. Figure 1b,c show the schematic diagrams of the basic metasurface unit and the corresponding geometric parameters. The main design concept of this work is to introduce a four-degree-of-freedom geometric encoding strategy into a single resonant structure. Unlike conventional metasurfaces that rely on one dominant geometric parameter for resonance tuning, the proposed unit incorporates four independently adjustable geometric parameters: the gap angle *θ*, outer ring radius *R*, scaling factor *S*, and ring width *W*.

By independently varying *θ*, *R*, *S*, and *W*, four geometric resonance-tuning pathways are established to regulate the resonance frequency and spectral response. These tuning pathways provide the basis for selecting encoded resonators with different resonance channels, thereby realizing broadband multichannel spectral coverage for terahertz molecular fingerprint sensing. The proposed structure can be fabricated by laser etching on aluminum foil. For the reference metasurface unit, the geometric parameters were set as follows: period *P* = 100 μm, gap angle *θ* = 140°, outer ring radius *R* = 40 μm, ring width *W* = 20 μm, scaling factor *S* = 1.0, and aluminum thickness *t* = 10 μm. The proposed structure is compatible with femtosecond-laser direct writing on thin aluminum foil. Lin et al. experimentally fabricated a substrate-free aperture array using a rigid holder and demonstrated THz biomolecular sensing through low-volume drop deposition [35]. A similar framed configuration and drop-drying protocol could reduce deformation and handling damage in the present design. It should be noted that *θ*, *R*, *S*, and *W* are fabrication-defined design degrees of freedom rather than real-time active tuning variables during THz-TDS measurement. In practical implementation, these parameters are predetermined in the CAD layout and then transferred to the aluminum foil by laser etching. Therefore, the proposed strategy provides designable resonance encoding through predesigned geometries, while real-time active tuning is not claimed in this work.

Full-wave electromagnetic simulations were performed using the finite-difference time-domain (FDTD) method in Lumerical FDTD Solutions 2020 R2 (Lumerical Inc., Vancouver, BC, Canada). Periodic boundary conditions were applied along the x- and y-directions to model an infinite array, while perfectly matched layers were used along the z-direction to absorb outgoing electromagnetic waves. A TE-polarized THz plane wave was normally incident along the z-direction, with the electric field polarized along the y-direction and the magnetic field along the x-direction, as indicated in Figure 1. To improve reproducibility, the computational unit cell was set to 100 μm × 100 μm × 200 μm. The source was located 80 μm below the metasurface reference plane, and the transmission monitor was located 50 μm above it. The lower PML-to-source distance and the upper monitor-to-PML distance were 20 and 50 μm, respectively. The exported spectra covered 0.3–2.3 THz with 1000 frequency points. An automatic nonuniform mesh with a mesh accuracy of 8 was used for all simulations, and the simulation time and auto-shutoff threshold were set to $1.0\times 10^{-10}$ s and ${1.0\times 10}^{-7}$, respectively. Aluminum was modeled using the Drude dispersion model [36]:

(1) $$\varepsilon \left(\omega \right)=\left(1-\frac{{\omega^{2}}_{p}}{\omega^{2}+\gamma^{2}}\right)+j\left(\frac{\gamma {\omega^{2}}_{p}}{\omega \left(\omega^{2}+\gamma^{2}\right)}\right)$$

where $\varepsilon \left(\omega \right)$ is the complex relative permittivity of aluminum, ω is the angular frequency of the incident THz wave, $\omega_{p}$ is the plasma frequency, γ is the damping rate, and $j=\sqrt{-1}$. According to the metal parameters used in the simulations, the plasma frequency and damping rate of aluminum were set to $\omega_{p}$ = 22.9 × $10^{15}$ $S^{-1}$ and *γ* = 0.92 × $10^{15}$ $S^{-1}$, respectively. The aluminum thickness was fixed at 10 μm in all simulations.

Although the present work is based on numerical simulations, the proposed device is intended to operate as a passive transmission-mode metasurface compatible with a conventional free-space THz-TDS system. In a future experiment, the chip can be placed at the sample position of a standard transmission setup and illuminated at normal incidence by a broadband linearly polarized THz pulse, with the electric field aligned with the resonator orientation used in the simulations. The transmitted time-domain waveforms of an empty aperture, an unloaded metasurface, and an analyte-loaded metasurface can be recorded under identical optical conditions. The empty-aperture signal serves as the reference, while comparison between the unloaded and analyte-loaded metasurface spectra is used to identify analyte-induced changes against the intrinsic structural response. Accordingly, the measurement can be performed with a single broadband THz beam using a conventional transmission geometry.

Before spectral extraction, all time-domain waveforms are baseline corrected and multiplied by the same Tukey window centered on the main peak of the reference pulse to suppress delayed echoes caused by multiple reflections. The windowed signals are then Fourier transformed, and the complex field transmission coefficient is calculated as $t\left(\omega \right)=\frac{E_{sam}\left(\omega \right)}{E_{ref}\left(\omega \right)}$, where $E_{sam}\left(\omega \right)$ and $E_{ref}\left(\omega \right)$ denote the Fourier-transformed sample and empty-aperture reference fields, respectively. The intensity transmittance is obtained as $T\left(\omega \right)=\;{\left|t\left(\omega \right)\right|}^{2}$, and the transmission attenuation is defined as $A\left(\omega \right)=1\;-\;T\left(\omega \right).$ Frequency points at which the reference amplitude is below 1% of its maximum are excluded to avoid unstable normalization. The resulting normalized transmission and attenuation spectra are used for the subsequent fingerprint analysis.

Unless otherwise specified, L-HYP was modeled as a homogeneous dispersive medium that completely filled the etched ring-shaped apertures throughout the 10 μm-thick aluminum layer and formed an additional 1 μm-thick overlayer above the metasurface. Its frequency-dependent absorption was assigned according to the literature-reported THz-TDS extinction spectrum of solid L-HYP [31,32,33,34], with the main absorption bands located near 0.73 and 1.17 THz. For future experimental implementation, trace biomolecules can be deposited onto the self-supporting metasurface by solution-based methods such as micropipette-assisted low-volume drop deposition followed by controlled drying followed by solvent evaporation; similar drop-drying strategies have been demonstrated for amino-acid detection using substrate-free THz metamaterial sensors [35]. The analyte concentration, solution volume, and deposition area can be adjusted to control the average areal density or equivalent film thickness. Because dried biomolecular films may still exhibit local nonuniformity, deposition uniformity and repeatability should be optimized in future experiments.

## 3. Results

### 3.1. Resonance Mechanism and Baseline Validation

**Figure 2:**
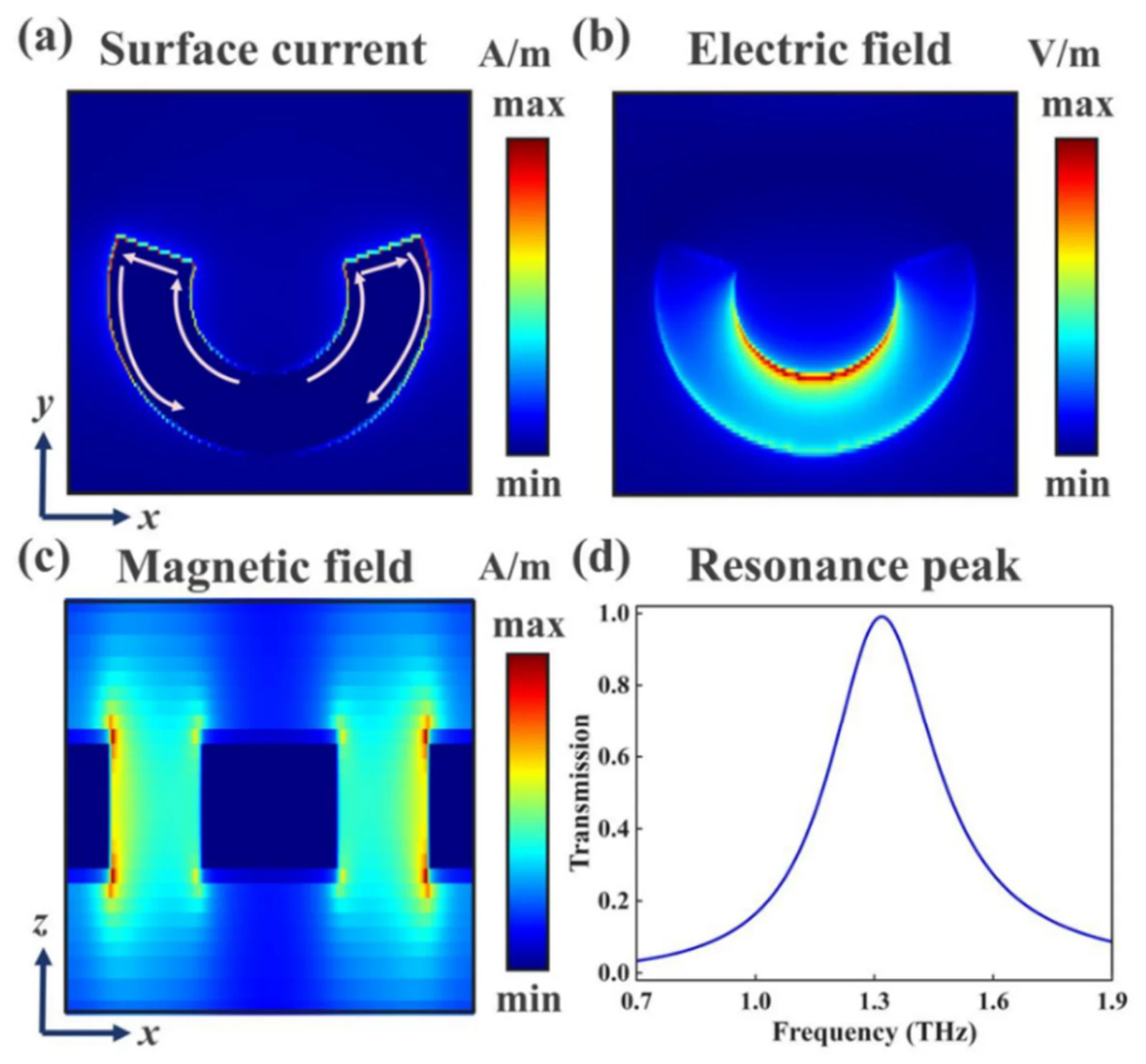
Mechanism and spectral response of the metasurface. (**a**) Simulated surface current distribution at the resonant frequency. (**b**) Corresponding electric field distribution. (**c**) Corresponding magnetic field distribution. (**d**) Simulated transmission spectrum of the metasurface sensor with a distinct resonant peak.

Figure 2 presents the electromagnetic responses and resonance mechanism of the metasurface. Figure 2a displays the surface-current distribution at resonance, where distinct circulating oscillations can be observed. The currents on the left and right sides flow in opposite directions, forming a magnetic-dipole-like response, as indicated by the arrows [37]. Figure 2b,c show the corresponding electric and magnetic field distributions, respectively. The field energy is strongly confined in the gaps and openings of the structure, providing the near-field basis for subsequent analyte–resonator interaction. Figure 2d plots the simulated transmission spectrum, where a prominent transmission peak appears at 1.32 THz with an intensity exceeding 99%.

The resonance mechanism can be qualitatively interpreted using a simplified equivalent LC model [36]. The resonance frequency is approximately expressed as

(2) $$f_{0}=\frac{1}{2\pi \sqrt{L_{eff}C_{eff}}}$$

where $L_{eff}$ is mainly associated with the effective circulating-current path, while $C_{eff}$ originates from charge accumulation and electric-field confinement around the gap and slit edges. Variations in the gap angle *θ*, outer ring radius *R*, scaling factor *S*, and ring width *W* modify the effective current path and capacitive coupling, thereby producing the geometry-dependent resonance shifts. This qualitative interpretation is consistent with the simulated surface-current and field distributions shown in Figure 2a–c. Because the proposed metasurface has a finite-thickness and dispersive three-dimensional configuration, the equivalent LC model is used to explain the underlying resonance mechanism, whereas the quantitative transmission spectra are obtained from full-wave FDTD simulations.

To verify the dielectric sensitivity of the basic resonant unit before constructing the four-degree-of-freedom encoded metasurface, a refractive-index sweep was first performed on the nominal split-ring structure. As the refractive index increased from 1.0 to 1.6, the resonance continuously red-shifted from 1.313 to 1.005 THz, yielding a sensitivity of 512.66 GHz/RIU with a linear fitting coefficient of $R^{2}=\;0.997\;$(Supplementary Figure S1 and Supplementary Note S1). This refractive-index sweep is used here as a baseline validation of the localized resonant field response, rather than as a standalone refractometric-sensing demonstration. This stable and nearly linear response confirms that the split-ring unit provides a reliable resonant building block for the subsequent geometric-encoding pathways. A concise fabrication-tolerance analysis was further performed and is summarized in Supplementary Figures S3 and S4. Under ±5% geometric deviations, all fitted *R*^2^ values remain higher than 0.997, indicating that the baseline refractometric response remains reasonably robust to moderate fabrication fluctuations.

### 3.2. Four-Channel Geometric Encoding Layout and Broadband Spectral Coverage

**Figure 3:**
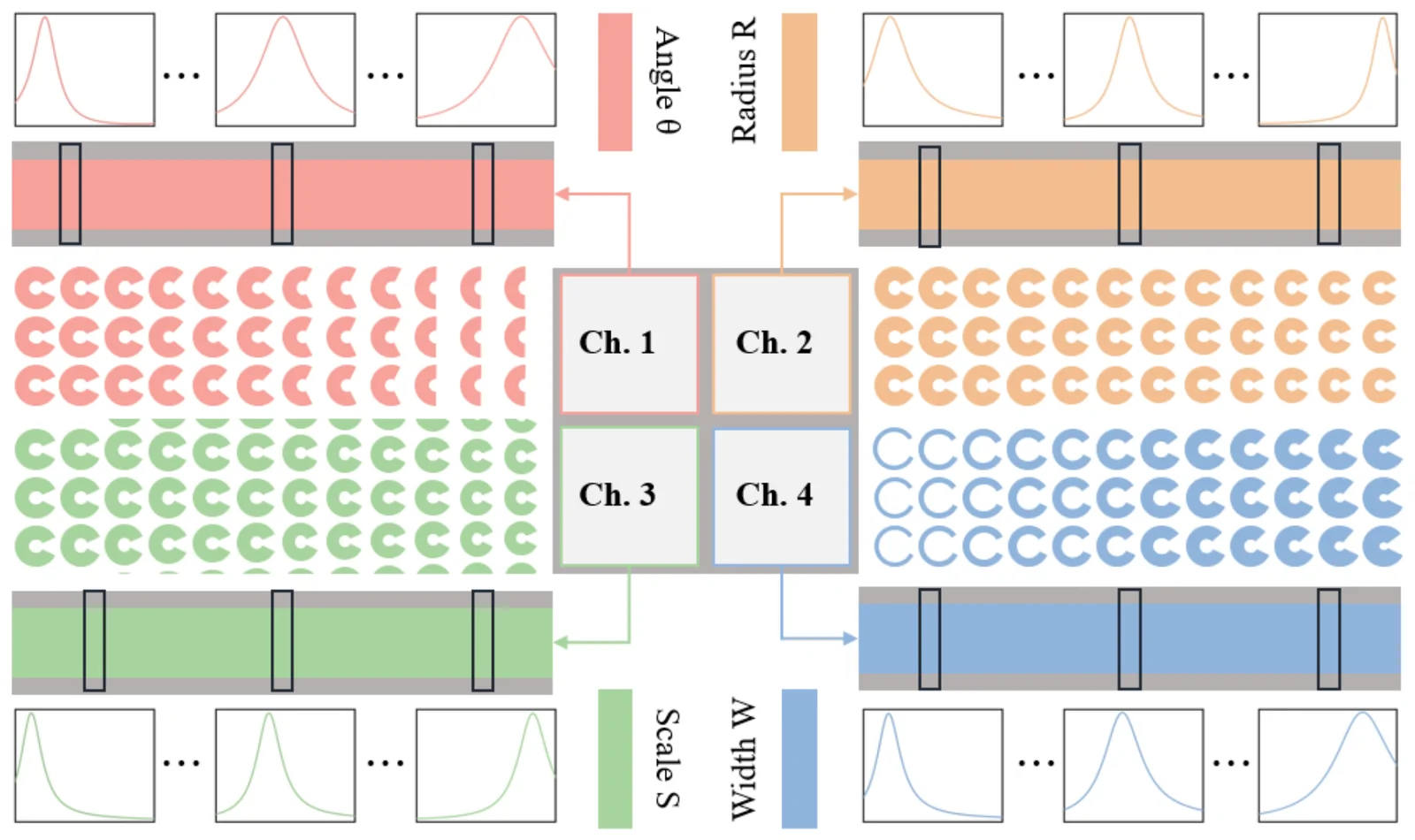
Schematic of the four-channel geometrically encoded metasurface. The 2 × 2 layout consists of four encoded resonator channels, denoted as Ch. 1–Ch. 4, which are associated with independent tuning pathways of *θ*, *R*, *S*, and *W*, respectively. The red, orange, green, and blue colors represent the *θ*-, *R*-, *S*-, and *W*-dependent tuning pathways, respectively, and the arrows indicate the correspondence between each encoded channel and its associated geometric-tuning pathway. The 2 × 2 arrangement schematically classifies the four independent geometric-tuning pathways and does not represent a physically coupled array of four different resonators.

To achieve broadband multichannel terahertz fingerprint sensing, four encoded resonators are spatially arranged in a 2 × 2 channel layout, as schematically shown in Figure 3. The four channels are assigned to independent geometric-tuning pathways associated with the gap angle *θ*, outer ring radius *R*, scaling factor *S*, and ring width *W*, respectively. Each parameter value corresponds to an independent periodic array of identical unit cells. The arrays are evaluated separately, and their transmission spectra are combined to construct the envelope of each geometric-tuning pathway. The 2 × 2 arrangement in Figure 3 is only a schematic classification of the four tuning pathways and does not represent a physically coupled array of four different resonators. In this way, each channel provides a distinct resonance response within the target spectral range. Compared with a single periodically repeated resonator, this discrete geometric-encoding strategy enables space-spectrum-correlated sampling and provides a compact route for broadband fingerprint enhancement.

The design workflow for the 2 × 2 encoded array is therefore organized as follows. First, the target L-HYP fingerprint bands near 0.73 THz and 1.17 THz are identified from the molecular extinction spectrum. Second, independent sweeps of *θ*, *R*, *S*, and *W* are performed while the other geometric parameters are fixed, so that each degree of freedom provides one single-parameter tuning pathway. Third, the resonance frequency $f_{0}$, bandwidth/FWHM, and average tuning coefficient $K\;=\;\left|\frac{{\Delta f}_{0}}{\Delta p}\right|$ are extracted from each sweep, where p denotes the corresponding geometric parameter. Fourth, encoded unit cells are selected from the four parametric gradients according to their spectral overlap with the target fingerprint windows and their ability to provide distinguishable channel responses. Finally, the selected units are assembled into the 2 × 2 spatial layout to achieve multiband frequency-selective spectral sampling under one broadband THz excitation in the simulation. Here, the term multichannel does not refer to multiple incident terahertz beams or multiple independent THz-TDS optical paths. Instead, it denotes a spatially encoded 2 × 2 metasurface layout in which different geometric unit cells provide discrete resonant bands. Under broadband THz-TDS illumination, these encoded resonators provide multiband frequency-selective spectral responses, so the target molecular fingerprint region can be sampled at multiple resonance bands in a compact metasurface design.

Compared with single-channel sensing, this spatially encoded multichannel strategy can improve recognition reliability because molecular identification is not judged from one isolated resonance alone. Responses from different encoded channels and from multiple L-HYP fingerprint bands, especially those near 0.73 THz and 1.17 THz, can be cross-validated, thereby reducing the possibility that nonspecific adsorption, sample-thickness fluctuation, environmental drift, or system noise is misinterpreted as a true molecular fingerprint signal.

**Figure 4:**
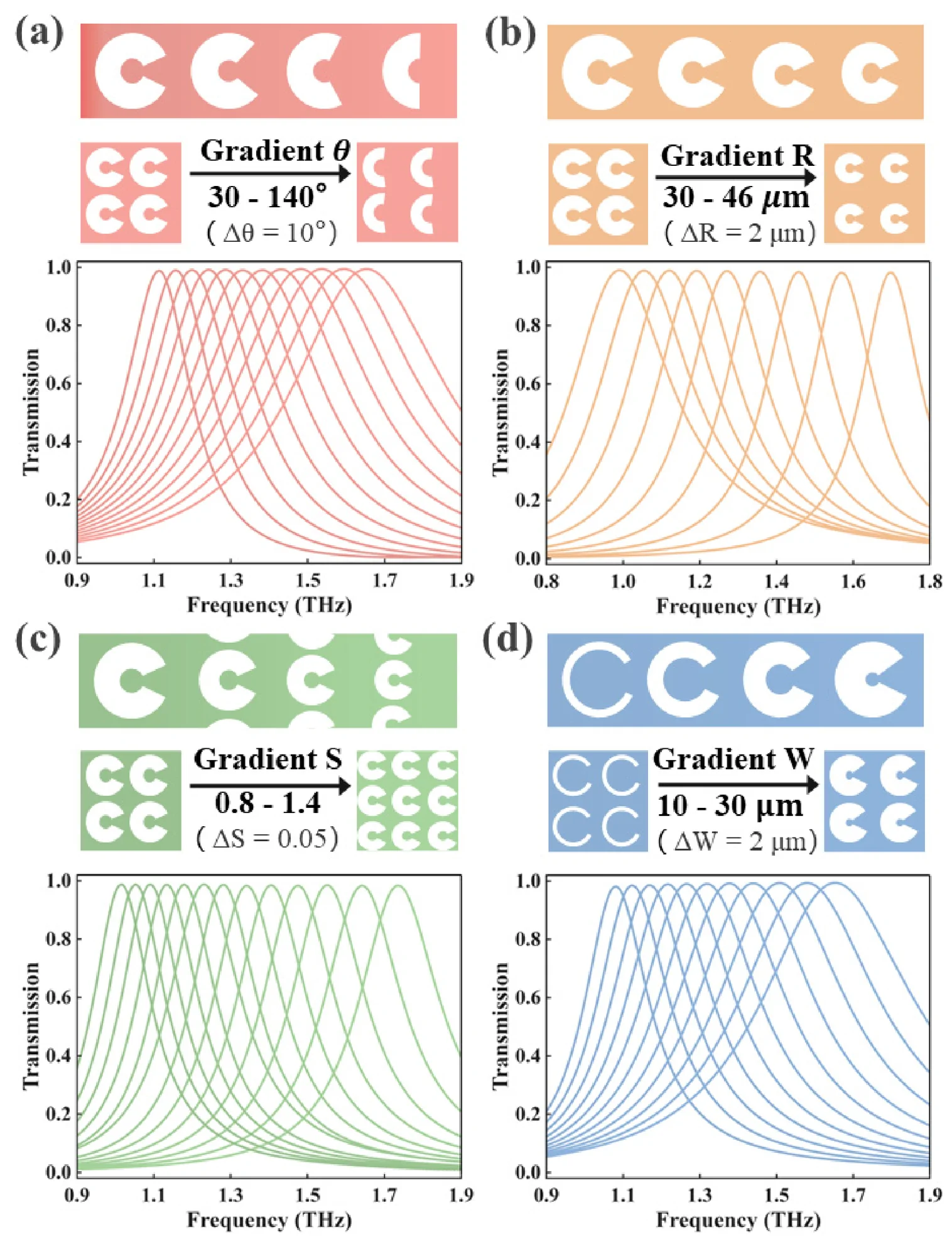
Transmission responses of the terahertz metasurface under four independent geometric-parameter sweeps. (**a**) Gap angle *θ* = 30–140° with Δ*θ* = 10°. (**b**) Outer ring radius *R* = 30–46 μm with Δ*R* = 2 μm. (**c**) Scaling factor *S* = 0.8–1.4 with Δ*S* = 0.05. (**d**) Ring width *W* = 10–30 μm with Δ*W* = 2 μm. Red, orange, green, and blue denote the *θ*-, *R*-, *S*-, and *W*-dependent parameter sweeps, respectively; within each panel, the color gradient represents successive values of the corresponding geometric parameter.

To realize broadband multiband responses within the target frequency range, independent parameter sweeps were performed for θ, *R*, *S*, and *W*. The resonance-tuning behavior induced by each geometric degree of freedom was systematically investigated, and the corresponding transmission spectra are presented in Figure 4. Figure 4a shows that increasing the gap angle θ produces a pronounced blue shift of the resonance while maintaining a stable transmission response, indicating that θ provides an efficient pathway for precise frequency regulation. Figure 4b illustrates the effect of the outer ring radius *R*; as *R* increases, the resonant peak shifts toward lower frequency, accompanied by regular changes in linewidth. Figure 4c shows that the scaling factor *S* provides global geometric scaling and drives the resonance smoothly across a broad frequency range. Figure 4d indicates that increasing the ring width *W* shifts the resonance toward higher frequency and modifies the transmission peak depth. To quantitatively support this design workflow, an internal single-parameter baseline comparison was performed within the same resonant architecture. The resonance-shift span Δf*,* average geometric shift coefficient $K\;=\;\left|\frac{{\Delta f}_{0}}{\Delta p}\right|$, and FWHM range were extracted for the *θ*-, *R*-, *S*-, and *W*-dependent pathways, as summarized in Table 1. This comparison clarifies the accessible spectral coverage and bandwidth behavior provided by each geometric pathway while avoiding ambiguity caused by cross-literature differences in materials, substrates, unit-cell topology, and bandwidth definitions. For example, in the *R*-dependent sweep, the resonance red-shifts from 1.698 to 0.991 THz as *R* increases from 30 to 46 μm, while the FWHM remains within 0.191–0.307 THz and the Q factor ranges from 3.23 to 8.90. These parametric sweeps provide a fabrication-defined design library from which encoded resonators can be selected according to their spectral overlap with the L-HYP fingerprint windows. Therefore, the four-degree-of-freedom geometric encoding strategy provides a practical basis for predesigned multiband spectral coverage and broadband fingerprint-enhanced sensing.

### 3.3. Biomolecular Sensing Performance and Fingerprint Enhancement Characteristics

**Figure 5:**
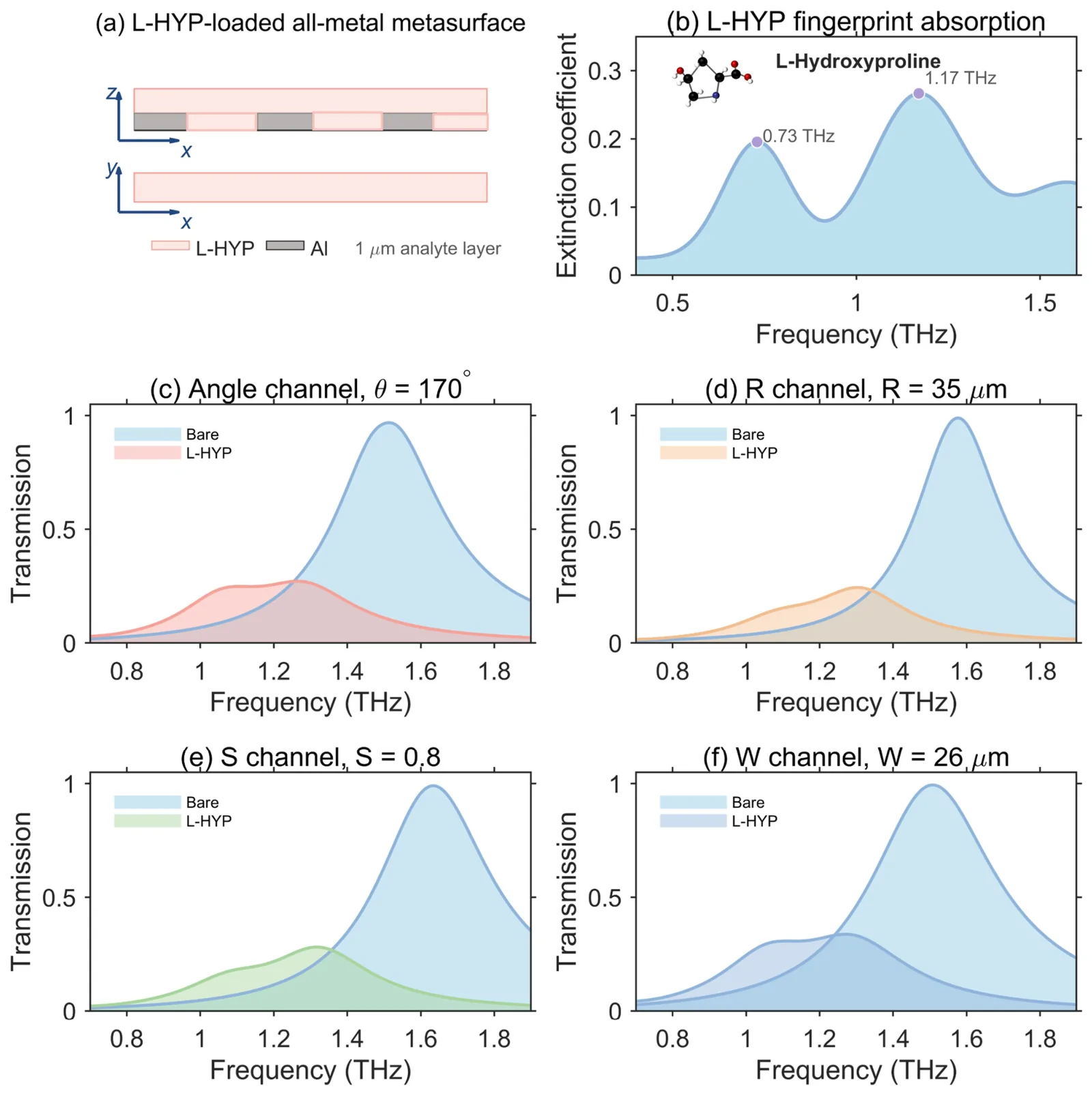
L-HYP-induced AIT-like fingerprint modulation in four encoded channels. (**a**) Schematic of the L-HYP-loaded metasurface, where light pink and gray denote the L-HYP analyte and aluminum, respectively. (**b**) Literature-reported THz fingerprint spectrum of L-HYP [31,32,33,34], with characteristic fingerprint bands near 0.73 and 1.17 THz. (**c**–**f**) Simulated transmission spectra before and after L-HYP loading for four representative encoded channels: (**c**) angle channel with *θ* = 170°, (**d**) radius channel with *R* = 35 μm, (**e**) scaling channel with *S* = 0.8, and (**f**) width channel with *W* = 26 μm. In (**c**–**f**), light blue denotes the bare metasurface, whereas red, orange, green, and blue denote the corresponding L-HYP-loaded *θ*, *R*, *S*, and *W* channels, respectively.

To evaluate the biomolecular fingerprint sensing capability of the proposed geometrically encoded metasurface, the L-HYP-loaded configuration was modeled as shown in Figure 5a. The L-HYP layer was directly placed onto the substrate-free resonator and the exposed slit, without inserting an additional dielectric spacer between the analyte and the field-concentrated metal gaps. This design follows substrate-free or aperture-type THz metamaterial sensing schemes, in which the absence of an additional dielectric spacer allows the analyte to interact more directly with the localized resonant near field [35]. Figure 5b shows the extinction coefficient spectrum of L-HYP, in which two characteristic absorption bands near 0.73 and 1.17 THz fall inside the geometrically encoded spectral range of the metasurface.

The AIT-like response is first examined using four representative encoded channels, as shown in Figure 5c–f. The angle channel (θ = 170°), radius channel (*R* = 35 μm), scaling channel (*S* = 0.8), and width channel (*W* = 26 μm) are designed so that their resonant responses overlap with the L-HYP fingerprint region. Before analyte loading, each channel provides a broad metasurface transmission resonance determined by its geometric parameter. After L-HYP loading, its narrow molecular absorption bands perturb the broader metasurface resonance through absorptive loss and dispersion. Reported THz-TDS and DFT studies attribute the bands around 0.73–0.76 THz and 1.13–1.17 THz to collective motions involving hydroxyl/carboxyl groups, pyrrolidine-ring vibrations, and intermolecular hydrogen bonding [31,32,33,34]. When these narrow molecular absorption bands spectrally overlap with the broader metasurface resonance and spatially overlap with the near-field region around the split-ring gaps and metal edges, the imaginary part of the molecular dielectric response increases localized loss, while the dispersive real part modifies the resonance line shape. As a result, weak molecular absorption is converted into a more visible AIT-like transmission modulation in the simulated spectra [38,39]. The different modulation amplitudes observed among the four channels indicate that the molecular readout is not determined by the analyte alone, but by the spectral overlap and near-field coupling strength between the encoded resonance and the L-HYP absorption band.

**Figure 6:**
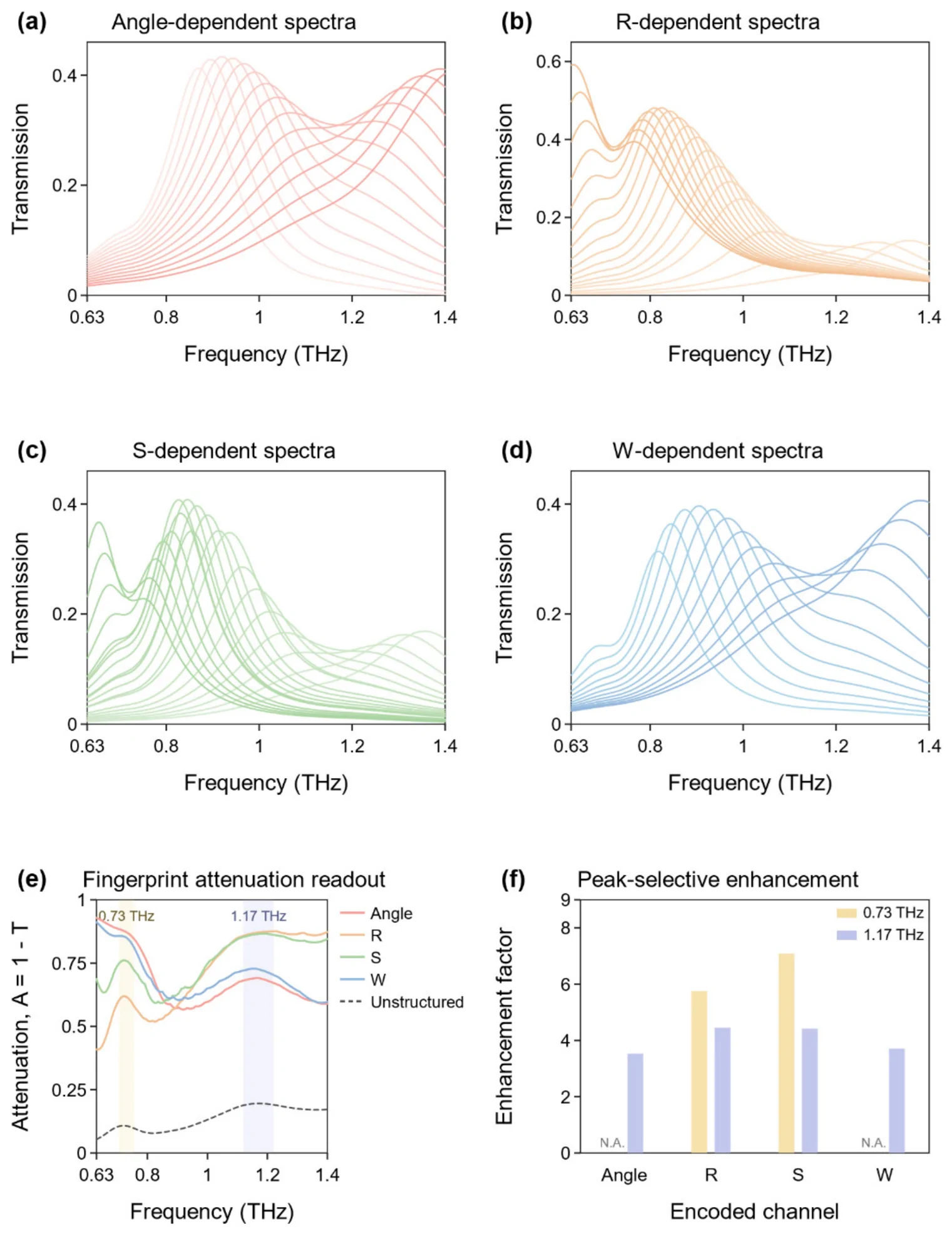
Broadband multichannel fingerprint enhancement and quantitative readout of the L-HYP-loaded metasurface. (**a**–**d**) L-HYP loaded transmission spectra obtained from the θ, R, S, and W geometric-tuning channels 0.63–1.4 THz. (**e**) Envelope-derived attenuation responses constructed by interpolating the resonance-maximum points of each channel, compared with that of an unstructured L-HYP reference layer of the same thickness. (**f**) Peak-selective fingerprint-enhancement factors at the L-HYP fingerprint frequencies of 0.73 and 1.17 THz, calculated as ${EF}_{c}$($f_{k}$) = $A_{meta,c}\left(f\right)/A_{ref}\left(f\right)$ The *θ* and W entries at 0.73 THz are marked as N.A. because the corresponding spectral features do not originate from their resonance-maximum envelopes and are therefore excluded from the enhancement-factor calculation.

Figure 5 demonstrates the local fingerprint-coupling mechanism, whereas Figure 6 extends this mechanism to broadband multichannel readout. The L-HYP-loaded transmission spectra obtained from the four geometric-tuning pathways are assembled in Figure 6a,d over 0.63–1.4 THz. In the θ-, *R*-, *S*-, and *W*-dependent spectra, the resonance envelopes sweep across the target fingerprint region, forming multiple frequency-selective spectral responses. This multichannel readout does not rely on multiple THz beams or multiple optical paths; instead, it uses spatially encoded resonators with different resonance bands to sample the molecular fingerprint region under one broadband THz excitation in simulation. Compared with a single isolated resonance, the L-HYP signal can be cross-validated by comparing its fingerprint responses across different encoded channels and jointly examining the variations in the two characteristic peaks at 0.73 and 1.17 THz, thereby reducing the likelihood that geometry-induced spectral changes or background perturbations are misinterpreted as molecular fingerprint signals.

To avoid ambiguity in data extraction, the envelope-extraction procedure and quantitative readout metrics are defined as follows. For each geometric-tuning channel c, the resonance maximum ($f_{r,c,j}$, $T_{r,c,j}$) was first extracted from each parameter-dependent transmission spectrum $T_{c,j}\left(f\right)$, where $c\in \left\{\;\theta ,R,S,W\right\}$ denotes the geometric-tuning channel, i denotes the i-th parameter value within that channel, f is the frequency, $f_{r,c,j}$ is the resonance frequency extracted from $T_{c,i}\left(f\right)$, and $T_{r,c,j}$ = $T_{r,i}\left(f_{r,c,j}\right)$ is the corresponding resonance transmission maximum. The upper transmission envelope $T_{env,c}\left(f\right)$ was then constructed by interpolating the set of resonance-maximum points:

(3) $$T_{env,c}\left(f\right)=\;{Interp\left\{\left(f_{r,c,i},T_{r,c,i}\right)\right\}}_{i=1}^{N_{c}}$$

where $Interp\left\{.\right\}$ denotes the interpolation operation and $N_{c}$ is the total number of parameter values used in channel c. The envelope-derived attenuation of the metasurface-loaded L-HYP layer was calculated as

(4) $$A_{meta,c}\left(f)=1-T_{env,c}(f\right)$$

where $A_{meta,c}$(f) denotes the attenuation obtained from channel c. For comparison, the attenuation of an unstructured L-HYP reference layer with the same analyte thickness was defined as

(5) $$A_{ref}(f)=1-T_{ref}(f)$$

where $T_{ref}$(f) and $A_{ref}$(f) denote the transmission and attenuation of the unstructured reference layer, respectively. The fingerprint-enhancement factor of channel c at the selected molecular fingerprint frequency $f_{k}$ was calculated as

(6) $${EF}_{c}\left(f_{k}\right)=\frac{A_{meta,c}\left(f\right)}{A_{ref}\left(f\right)}$$

where $f_{k}$ denotes the k-th target molecular fingerprint frequency and ${EF}_{c}$($f_{k}$) is the corresponding peak-selective enhancement factor. For L-HYP, $f_{1}$ = 0.73 THz and $f_{2}$ = 1.17 THz.

A fingerprint readout was considered valid only when the target molecular frequency lay within the resonance-sweeping range of the corresponding geometric-tuning channel and the attenuation at that frequency was derived from the interpolated trajectory of the resonance maxima. Features originating from non-resonant portions of the individual spectra were not considered part of the resonance envelope and were excluded from the enhancement-factor calculation. According to this unified criterion, the attenuation features of the R and S channels near 0.73 THz are located on their respective resonance-maximum envelopes, yielding enhancement factors of 5.76 and 7.09, respectively. In contrast, the features observed in the *θ* and W-dependent spectra near 0.73 THz do not originate from their resonance-maximum trajectories and are therefore marked as N.A. in Figure 6f. At 1.17 THz, all four geometric-tuning channels satisfy the envelope criterion, giving enhancement factors of 3.53, 4.45, 4.42, and 3.71 for the *θ*, R, *S*, and W channels, respectively. These results demonstrate that the proposed metasurface provides both qualitative fingerprint recognition and a quantifiable channel-selective enhancement response. To examine whether the four-degree-of-freedom geometric-encoding strategy is applicable beyond L-HYP, an additional four-channel simulation was performed for L-glutamic acid (L-Glu), which has a literature-reported fingerprint peak at 1.21 THz [40]. As summarized in Supplementary Figure S5, the envelope-derived enhancement factors at 1.21 THz are 3.3, 4.4, 4.3, and 3.4 for the *θ*, R, *S*, and W channels, respectively, relative to the unstructured L-Glu reference. These results further support the applicability of the geometric-encoding strategy to another analyte with a fingerprint band within the designed spectral range.

To evaluate the influence of realistic deposition nonuniformity, additional FDTD simulations were performed using the *S*-dependent pathway as a representative case. The complete *S* = 0.8–1.4 sweep was recalculated because deposition-induced resonance shifts may change the resonant unit contributing to the envelope at 1.17 THz. Partial slit filling, variable overlayer thickness, internal air voids, and a combined nonuniform condition were examined. The investigated cases retain 66.3–101.0% of the baseline envelope attenuation at 1.17 THz, while the combined nonuniform condition retains 72.8%. Because the unstructured reference remains unchanged, these percentages also represent the relative retention of the corresponding enhancement factor. Therefore, deposition nonuniformity reduces the response magnitude but does not eliminate the 1.17 THz fingerprint readout. Detailed models and results are provided in Supplementary Note S5 and Supplementary Figures S6 and S7.

These results indicate that the four-degree-of-freedom encoded metasurface enhances the L-HYP fingerprint response through complementary local and broadband readouts: AIT-like spectral modulation when encoded resonances overlap intrinsic molecular absorption bands, and envelope-derived attenuation contrast across the geometric sweep. The additional L-Glu simulation in Supplementary Figure S5 further suggests that the same encoding strategy can be applied to another analyte with a fingerprint band located within the designed spectral window. Therefore, the current results provide a simulation-based proof-of-concept for broadband multichannel fingerprint enhancement, while broader analyte selectivity and practical detection performance should be further validated in future experimental studies.

As summarized in Table 2, the proposed metasurface achieves a refractive-index sensitivity of 512.66 GHz/RIU, an average FOM of 2.14 RIU^−1^, spectral coverage of 0.6–1.4 THz, and fingerprint-enhancement factors of 3.53–7.09. Although some high-Q or specialized structures exhibit higher FOM or fingerprint-enhancement values, the present design combines four independent geometric-tuning pathways, broadband fingerprint accessibility, and a single-layer substrate-free aluminum configuration. Its principal advantage therefore lies in the balance among sensing performance, broadband coverage, multichannel fingerprint matching, and fabrication simplicity rather than the maximization of one individual metric.

## 4. Conclusions

In this work, a pixelated-gradient terahertz metasurface sensor with four-degree-of-freedom geometric encoding is designed and verified for broadband multichannel fingerprint sensing. By independently regulating the gap angle θ, outer ring radius R, scaling factor S, and ring width W within a 2 × 2 pixelated layout, geometrically encoded multiband resonances are obtained over the target 0.6–1.4 THz range. The aluminum self-supporting and substrate-free structure improves localized field enhancement. For L-HYP detection, the selected encoded resonators produce AIT-like spectral modulation near the molecular fingerprint bands, while the broadband spectral families provide envelope-derived attenuation-contrast enhancement relative to an unstructured analyte reference of the same thickness. These results confirm that the proposed metasurface does not merely tune resonant frequencies, but establishes a channel-selective molecular fingerprint amplification mechanism. Rather than relying on dynamic switching, the proposed strategy exploits designable multiband spectral coverage, frequency-selective multiplexing, and near-field molecular coupling to improve fingerprint recognition reliability. Featuring compact structure, simple fabrication, low cost, and favorable on-chip integration compatibility, this sensor provides a feasible route for terahertz trace biomolecule detection, broadband fingerprint recognition, collagen-metabolism-related biomarker monitoring, and multicomponent biochemical analysis.

## Figures and Tables

**Table 1:** Quantitative comparison of single-parameter tuning baselines and the proposed four-degree-of-freedom encoded design extracted from Figure 4. Δf denotes the resonance-frequency tuning span, and *K* = |Δ$f_{0}$/Δ*p*| is the average tuning coefficient.

Strategy	Parameter Range	$f_{0}$ Range (THz)	Δf (GHz)	Average *K*	FWHM Range (THz)	Valid L-HYP Readout
*θ*-only baseline	*θ* = 30–140°	1.113–1.653	540.5	4.91 GHz/°	0.203–0.475	1.17 THz
*R*-only baseline	*R* = 30–46 μm	0.991–1.698	707.2	44.20 GHz/μm	0.191– 0.307	0.73 and 1.17 THz
*S*-only baseline	*S* = 0.8–1.4	1.017–1.736	718.7	1197.84 GHz/unit *S*	0.183–0.257	0.73 and 1.17 THz
*W*-only baseline	*W* = 10–30 μm	1.081–1.653	572.1	28.60 GHz/μm	0.204–0.475	1.17 THz
Four-DOF encoded design	*θ/R/S/W* integrated	0.991–1.736	744.7	multiple pathways	0.183–0.475	0.73 and 1.17 THz

Note: This internal baseline comparison avoids ambiguity caused by cross-literature differences in materials, substrates, operating frequencies, unit-cell topologies, and bandwidth definitions.

**Table 2:** Quantitative comparison of the proposed metasurface with representative recently reported THz metasurface sensors.

Reference	Tuning/Encoding Method	Spectral Coverage	RI Sensitivity	FOM	Fingerprint Enhancement	Fabrication Complexity
Pan et al. (2025) [19]	Angle scanning + geometric scaling	1.0–2.8 THz	500 GHz/RIU	N.R.	5.74–6.47–fold	Copper/LCP and photolithography
Han et al. (2024) [27]	Radius-gradient pixel array	1.5–2.3 THz	263.9 GHz/RIU	5.03 RIU^−1^	10.4–fold	Metal/quartz and photolithography
Lin et al. (2024) [35]	Asymmetry-controlled qBIC	Fixed dipole/qBIC resonances	447/860 GHz/RIU	2.9/10.5 RIU^−1^	N.R.	Single-layer aluminum foil and femtosecond laser
Yang et al. (2025) [41]	Pixelated quasi-BIC	N.R.	N.R.	N.R.	≈70% signal enhancement	Dielectric multilayer/sandwich structure
Zhu et al. (2024) [42]	Geometry and incident-angle multiplexing	0.48–0.58 THz	N.R.	N.R.	≈120–fold	Ultrathin patterned metal-groove array
Present work	Four *θ*/*R*/*S*/*W* tuning pathways	0.6–1.4 THz	512.66 GHz/RIU	2.14 RIU^−1^	3.53–7.09–fold	Single-layer substrate-free aluminum foil and laser processing

Note: N.R., not reported.

## Data Availability

The data presented in this study are available on request from the corresponding author due to the absence of a dedicated public data repository for these simulation datasets.

## Supplementary Materials

The following supporting information can be downloaded at: https://www.mdpi.com/article/10.3390/nano16171059/s1, Figure S1: Refractive-index sensing performance of the proposed terahertz metasurface; Figure S2: Quantitative FWHM and Q factor extraction for the four geometric sweeps; Figure S3: Linear fitting of resonance frequency versus analyte refractive index under fabrication deviations of *θ*, *R*, *W*, *S*, and g; Figure S4: Relative sensitivity change compared with the nominal structure under ±2% and ±5% fabrication deviations; Figure S5: Additional L-glutamic acid (L-Glu) full-sweep fingerprint-sensing simulation; Figure S6: Sensitivity of the S-encoded transmission spectra to nonuniform analyte deposition; Figure S7: Sensitivity of the envelope-derived attenuation at 1.17 THz to nonuniform analyte deposition.

## Abbreviations

The following abbreviations are used in this manuscript:

THz	Terahertz
FDTD	Finite-Difference Time-Domain
FWHM	Full Width at Half Maximum
Q-factor	Quality Factor
FOM	Figure of Merit
L-HYP	L-Hydroxyproline
L-GLU	L-Glutamic Acid
THz-TDS	Terahertz Time-Domain Spectroscopy
