# Supplementary material for: Terahertz Metasurface with Four-Degree-of-Freedom Geometric Encoding for Broadband Multichannel Fingerprint Sensing
Source: Nanomaterials (Basel). 2026 Aug 26;16(17):1059. doi: 10.3390/nano16171059 (PMC13566993; doi:10.3390/nano16171059)
Supplement: Supplementary file 1 [file nanomaterials-16-01059-s001.zip › nanomaterials-4510220-supplementary.pdf]

Supplementary Information

This supplementary file provides the refractive-index baseline sensing results, quantitative FWHM/quality (Q) factor extraction for the four geometric sweeps, fabrication-tolerance analysis, and an additional L-glutamic acid (L-Glu) fingerprint-sensing simulation supporting the revised manuscript.

Supplementary Note S1. Refractive-index sensing and FOM extraction

The refractive-index sensing performance was evaluated by loading uniform dielectric overlayers with n = 1.0–1.6 (interval 0.1) on the nominal metasurface while keeping all structural parameters unchanged. For each refractive index, the transmission spectrum was calculated and the main resonance frequency was extracted. The refractive-index sensitivity was defined as $SR = \Delta f/\Delta n$. As shown in Supplementary Fig. S1(a,b), the resonance red-shifts from 1.313 to 1.005 THz and gives $SR = 0.5127$ THz/RIU with $R^2 = 0.9971$.

To quantify the baseline sensing performance, the linewidth and Q factor were extracted from the same simulated spectra. The Q factor increases from 4.18 to 5.22 as the refractive index increases, with a mean value of 4.72. The FOM was calculated as $FOM = SR/FWHM$, where S is the refractive-index sensitivity. The FOM ranges from 1.63 to 2.67 RIU^{-1}, with an average value of 2.14 RIU^{-1}. These results confirm a stable refractometric response of the nominal split-ring unit.

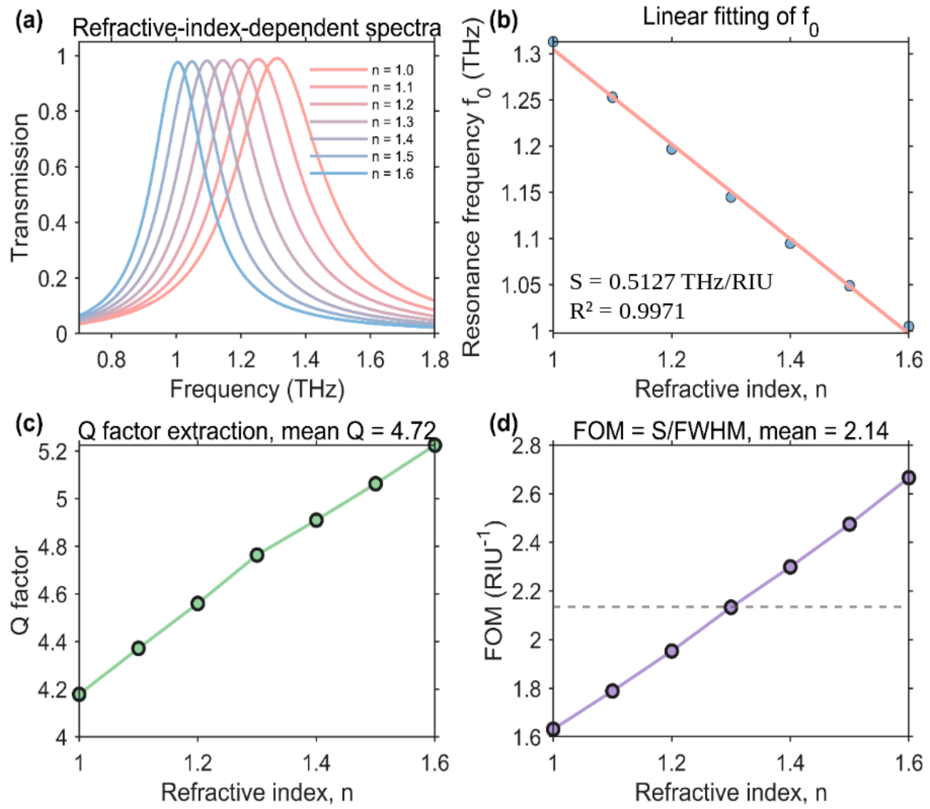

Supplementary Figure S1. Refractive-index sensing performance of the proposed terahertz metasurface. (a) Simulated transmission spectra under different refractive indices from n = 1.0 to n = 1.6. (b) Extracted resonance frequency as a function of refractive index and corresponding linear fit. (c) Extracted Q factor under different refractive indices. (d) Figure of merit ($FOM = SR/FWHM$) as a function of refractive index.

Supplementary Note S2. Geometric-sweep linewidth and Q factor extraction

The resonance frequency, FWHM, and Q factor were extracted from the dominant resonance in each geometric sweep. Supplementary Fig. S2 summarizes the linewidth and Q factor trends when θ, R, S, and W are varied.

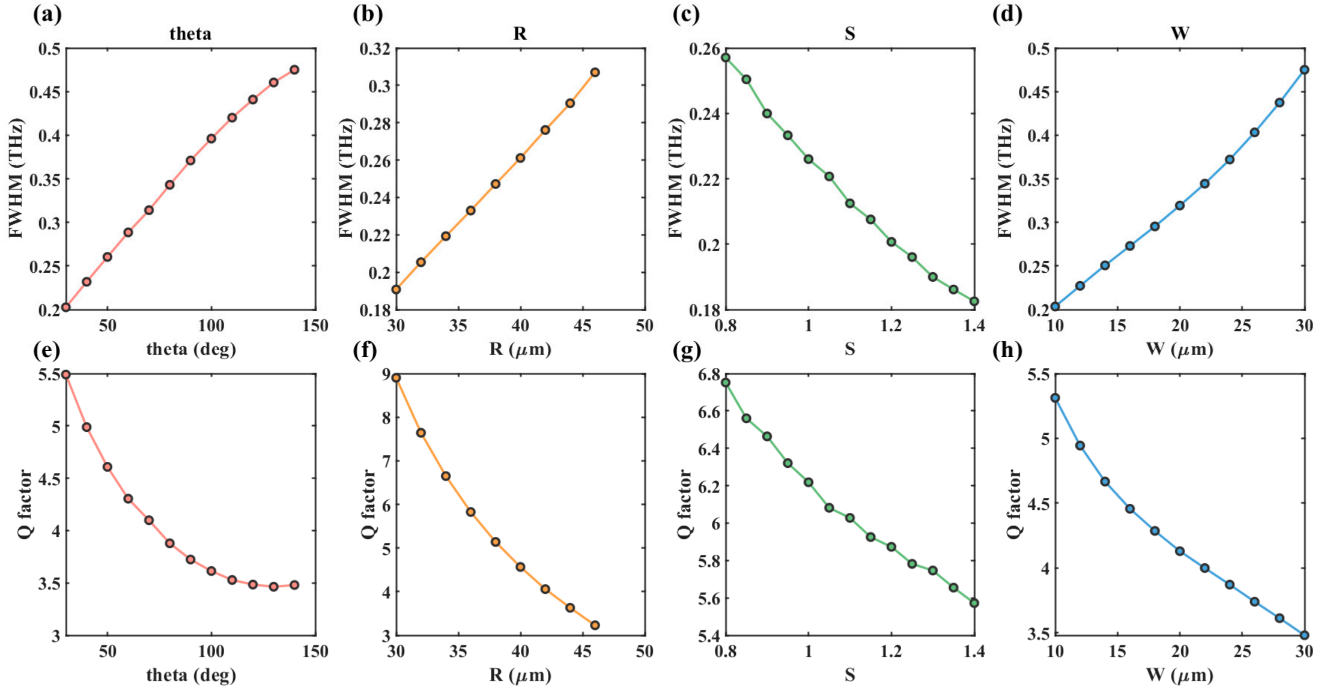

Supplementary Figure S2. Quantitative FWHM and Q factor extraction for the four geometric sweeps.

Supplementary Note S3. Fabrication-tolerance analysis

A concise fabrication-tolerance analysis was performed to assess the robustness of the refractive-index response under realistic geometric deviations. The nominal geometry and 20 perturbed geometries were simulated by independently varying θ, R, W, S, and the gap opening g by ±2% and ±5%, with n = 1.0, 1.2, 1.4, and 1.6 calculated for each geometry. In these simulations, the analyte was assigned to both the upper analyte layer and the exposed etched ring, matching the analyte-loaded configuration used in the sensing simulations.

The nominal structure gives a sensitivity of 0.51502 THz/RIU with $R^2 = 0.997$ and a mean Q factor of 4.69 in this dataset. Under ±5% deviations, the sensitivity variation ranges from −3.36% to +2.91% for θ, − 3.94% to +3.35% for R, − 2.04% to +1.02% for W, − 5.98% to +4.96% for S, and − 5.83% to +9.77% for g. All fitted R^2 values are higher than 0.9971. These results indicate that the refractive-index response remains approximately linear under moderate fabrication fluctuations, while the gap opening g and the geometric scaling factor S are the most influential dimensions and should be controlled carefully during fabrication.

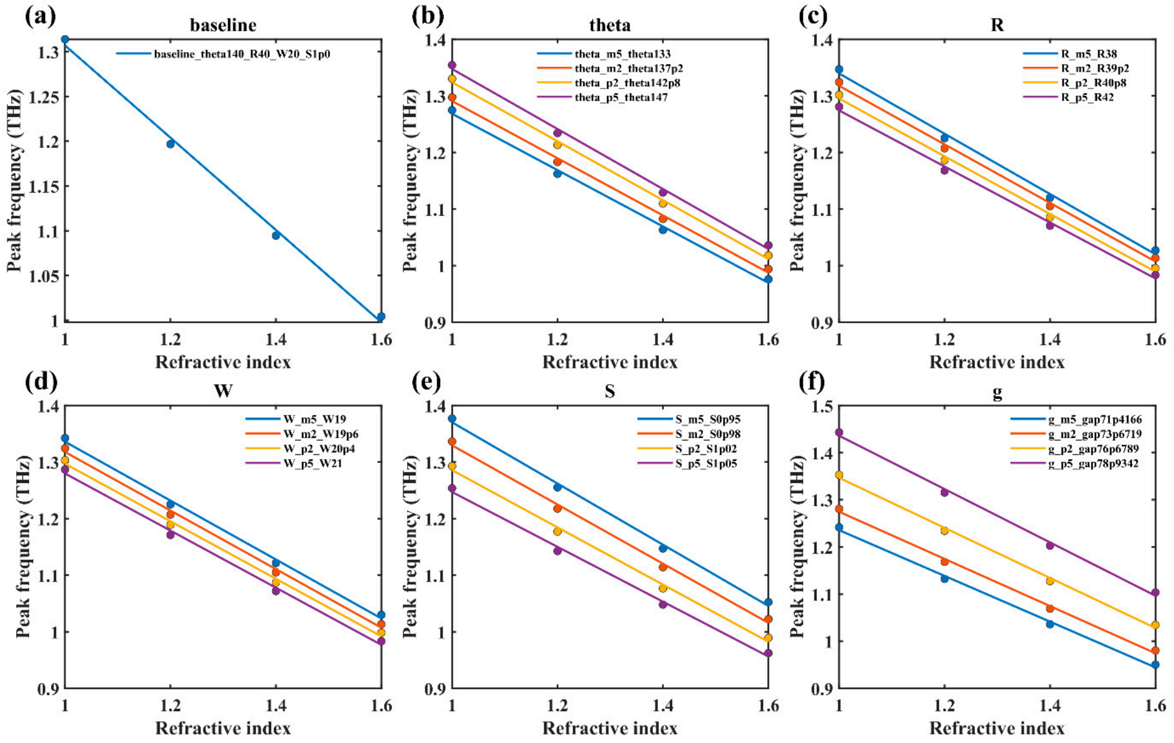

Supplementary Figure S3. Linear fitting of resonance frequency versus analyte refractive index under fabrication deviations of θ, R, W, S, and g.

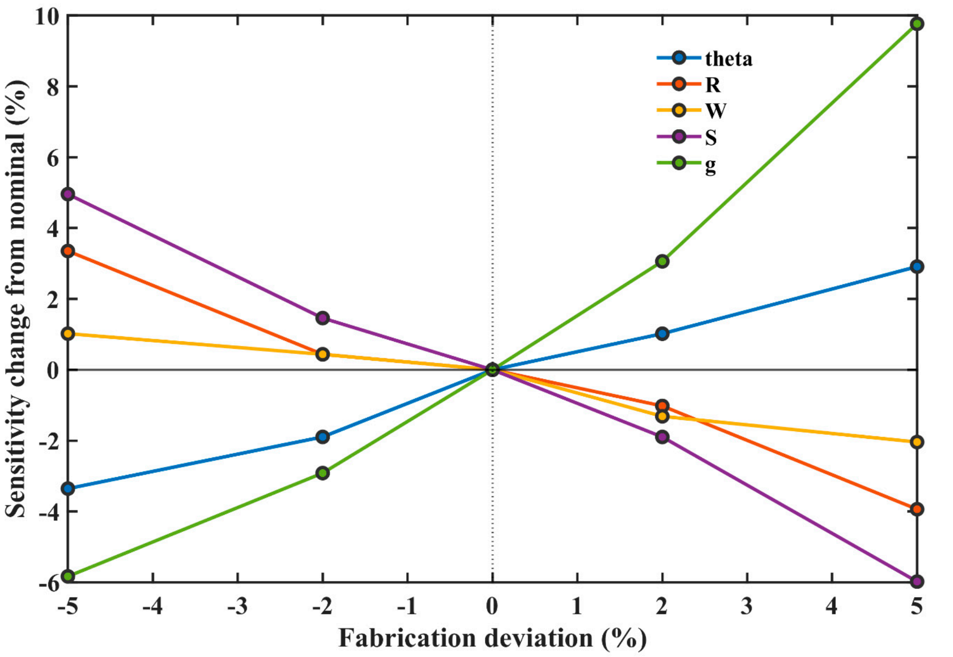

Supplementary Figure S4. Relative sensitivity change compared with the nominal structure under ±2% and ±5% fabrication deviations

Supplementary Note S4. Additional L-Glu full-sweep fingerprint-sensing simulation

To further evaluate whether the proposed four-degree-of-freedom geometrical encoding strategy is applicable beyond L-hydroxyproline (L-HYP), an additional analyte, L-Glu, was simulated. The literature-derived L-Glu dielectric response, extracted from reported THz-TDS data [40], contains a pronounced fingerprint peak at 1.21 THz, which lies within the encoded 0.7–1.4 THz spectral window. The same full θ-, R-, S-, and W-dependent sweep sets were calculated, and the L-Glu material was assigned to both the upper analyte layer and the exposed etched slit. An unstructured L-Glu reference with the same analyte loading was also simulated.

The channel readout was obtained using the same envelope-based treatment as in the L-HYP analysis. Specifically, the upper transmission envelope of each full sweep was converted into attenuation, A = 1 − T, and the enhancement factor was calculated as A_{meta}/A_{ref} at the L-Glu fingerprint peak of 1.21 THz. As summarized in Supplementary Fig. S5, the θ, R, S, and W channels give enhancement factors of 3.3, 4.4, 4.3, and 3.4, respectively. These results show that the encoded metasurface can enhance the fingerprint response of an additional analyte whose characteristic band lies within the geometrically encoded spectral range.

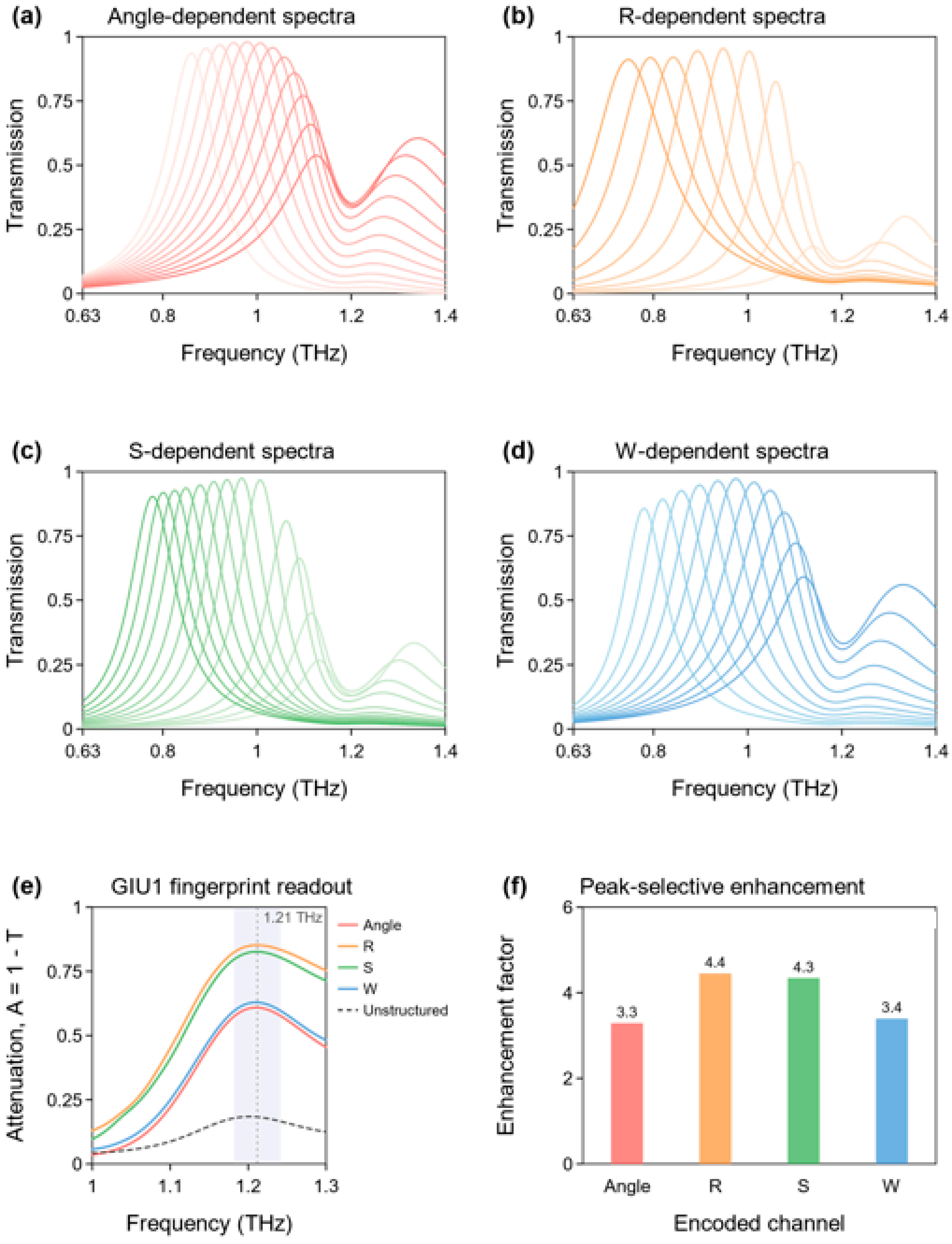

Supplementary Figure S5. Additional L-glutamic acid (L-Glu) full-sweep fingerprint-sensing simulation. (a–d) L-Glu-loaded transmission spectra obtained from the θ, R, S, and W tuning pathways over 0.63–1.4 THz. (e) Envelope-derived attenuation readout, A = 1 − T, compared with the unstructured L-Glu reference. The dashed vertical line marks the L-Glu fingerprint peak at 1.21 THz. (f) Peak-selective enhancement factors at 1.21 THz for the four encoded channels, calculated relative to the unstructured L-Glu reference.

Supplementary Note S5. Sensitivity to nonuniform analyte deposition

To evaluate the influence of realistic nonuniform deposition in the gap region, a sensitivity analysis was performed using the S-encoded pathway as a representative case. The scaling factor was swept from $S = 0.8$ to 1.4 in increments of 0.1, while $\theta = 140°$, $R = 35$ µm, $W = 15$ µm, and $P = 100$ µm were used as the reference geometric parameters. The baseline model comprised complete filling of the 10-µm-deep slit and a uniform 1 µm analyte overlayer. Three types of deposition nonuniformity were then considered: (i) partial slit filling of 25%, 50%, and 75% of the slit depth; (ii) overlayer thicknesses of 0, 0.5, and 2 µm; and (iii) centered internal air voids occupying 25% and 50% of the filled slit volume. A combined nonuniform case with 50% slit filling, a 0.5-µm overlayer, and a 25% internal void was also simulated.

The resulting S-dependent transmission spectra are shown in Supplementary Fig. S6. Reduced slit filling and internal air voids modify the resonance amplitude and lineshape, but the sequence of tunable resonances remains distinguishable over 0.7–1.5 THz. For quantitative comparison at the L-HYP fingerprint band of 1.17 THz, the upper transmission envelope was converted to attenuation, $A = 1 - T$, and the retained attenuation was defined as $\rho = A_{nonuniform}/A_{baseline} \times 100\%$. As summarized in Supplementary Fig. S7, slit filling fractions of 75%, 50%, and 25% retain 93.2%, 86.2%, and 66.3% of the baseline attenuation, respectively. Internal void fractions of 25% and 50% retain 93.3% and 86.2%, whereas overlayer thicknesses of 0, 0.5, and 2 µm retain 97.7%, 99.5%, and 101.0%, respectively. Even the combined nonuniform case retains 72.8% of the baseline response. These results indicate that incomplete gap filling and trapped air primarily reduce the response magnitude, while moderate overlayer-thickness variation has a smaller influence. Therefore, the homogeneous gap-filling assumption represents an upper-bound idealization with respect to incomplete filling and void formation, but the 1.17-THz fingerprint response remains observable under the examined nonuniform deposition conditions.

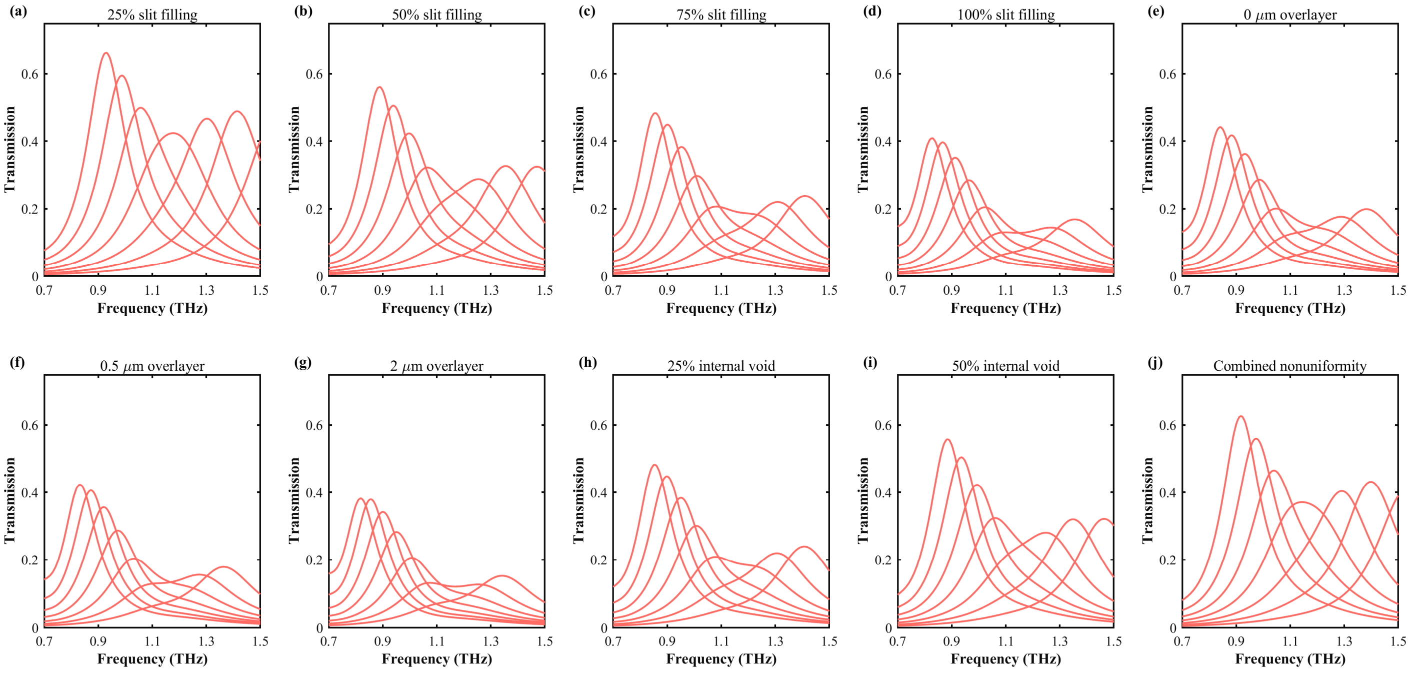

Supplementary Figure S6. Sensitivity of the *S*-encoded transmission spectra to nonuniform analyte deposition. The scaling factor is varied from $S = 0.8$ to 1.4. (a–d) Slit filling fractions of 25%, 50%, 75%, and 100%, respectively. (e–g) Uniform analyte-overlayer thicknesses of 0, 0.5, and 2 µm, respectively. (h,i) Centered internal air-void fractions of 25% and 50%, respectively. (j) Combined nonuniform condition with 50% slit filling, a 0.5-µm overlayer, and a 25% internal void.

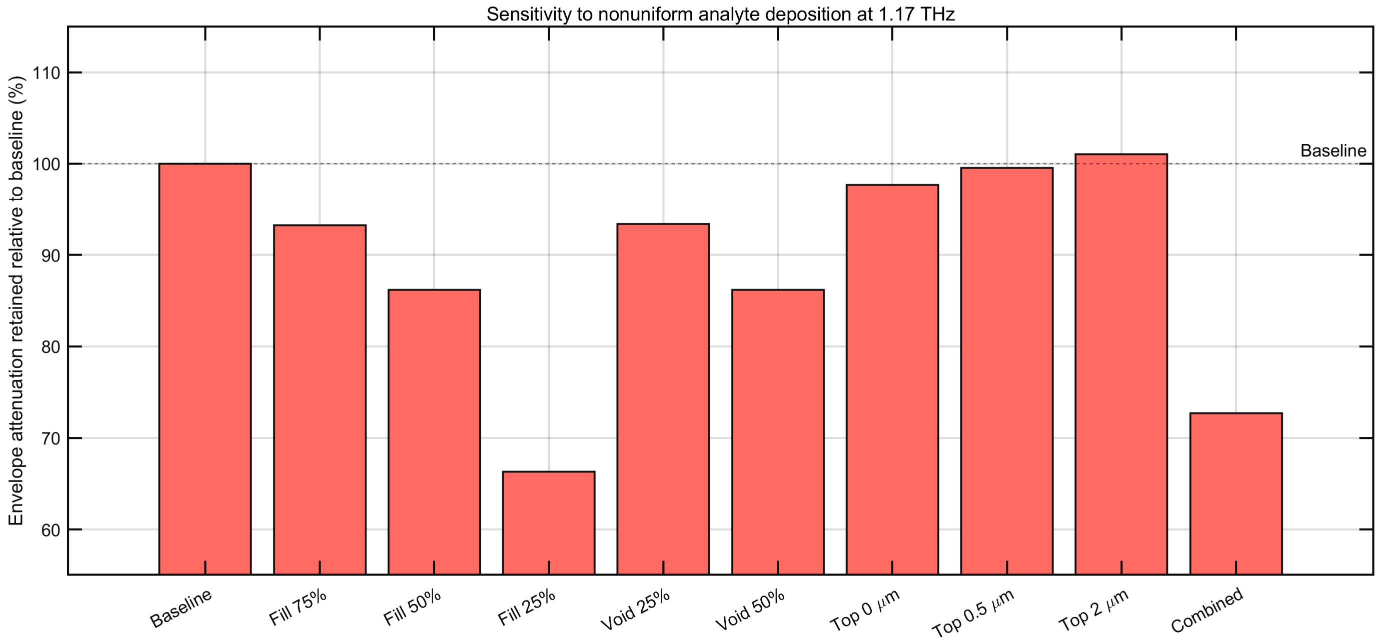

Supplementary Figure S7. Sensitivity of the envelope-derived attenuation at 1.17 THz to nonuniform analyte deposition. Each bar gives the attenuation retained relative to the baseline model with complete 10-µm slit filling and a uniform 1-µm overlayer. The dashed horizontal line denotes the 100% baseline level.
